# Editorial: Targeted metagenomics in pathogen detection

**DOI:** 10.3389/fcimb.2025.1612802

**Published:** 2025-05-16

**Authors:** Jiemin Zhou, Edwin Kamau, Qing Wei

**Affiliations:** ^1^ Medical Marketing Center, Vision Medicals Co, Ltd, Guangzhou, China; ^2^ Department of Pathology and Area Laboratory Services, Tripler Army Medical Center, Honolulu, HI, United States; ^3^ Shanghai Cinopath Medical Laboratory Co., Kindstar Globalgene Technology Inc., Shanghai, China; ^4^ Department of Research and Development, Kindstar Global Precision Medicine Institute, Wuhan, China

**Keywords:** metagenomics, next generation sequencing, clinical microbiology, infectious dieseases, targeted metagenomics

## Introduction

Infectious diseases are the leading cause of morbidity and mortality worldwide, accounting for approximately 25.5% of global deaths (Diseases and Injuries, 2020). The low sensitivity of conventional diagnostic methods and long turnaround times pose significant challenges for timely and accurate diagnosis, which is critical for improving patient prognosis. Unbiased metagenomics, a high-throughput and non-targeted technology used to analyze all genomic information in a sample, has been widely used to diagnose various infections, such as bloodstream infections, abdominal cavity infections, and central nervous system infections. Metagenomics has become a promising detection method for infectious diseases. While targeted metagenomics, a modified technique, focuses on sequencing specific genes or microbial communities, providing more focused data on selected regions or species, and it allows for the selective enrichment and sequencing of specific microbial species or communities within complex samples, such as those found in clinical settings. This technique is particularly useful when traditional culture-based methods fail to detect the causative pathogen or when multiple pathogens are present in the same sample. Therefore, targeted metagenomics has the potential to revolutionize the diagnosis and treatment of infectious diseases in clinical settings, providing a more personalized approach to healthcare.

## Goals of targeted metagenomics

The primary objective of targeted metagenomics is to identify and characterize microbial communities in clinical samples—such as blood, urine, and sputum—to aid in the diagnosis and treatment of infectious diseases. Additionally, targeted metagenomics has applications in other fields, including agriculture and forensic medicine. This technique seeks to lower the cost of conventional metagenomics, which has been proposed as a method to detect all potential pathogens in clinical samples. In addition, targeted metagenomics seeks to improve the performance of current approaches, such as 16S/18S rRNA-based amplicon sequencing and custom-designed primer pools. Establishing guidelines to standardize the workflow of targeted metagenomics across different sequencing platforms is also a key objective. Ultimately, targeted metagenomics aims to offer an affordable, accurate, and fast approach for precise pathogen detection.

## Clinical applications of targeted metagenomics

Immunocompromised patients are more susceptible to infections by rare (Zhan et al., 2021), regional (Ramirez et al., 2020), and emerging (El Zein et al., 2020; Fishman, 2023) pathogens, posing significant challenges for the clinical application of targeted metagenomics. Liu et al. retrospectively enrolled 546 immunocompetent and immunocompromised patients with suspected community-acquired pneumonia to evaluate the performance of metagenomics and targeted metagenomics. The total coincidence rate of targeted metagenomics was much higher than that of metagenomics, with final comprehensive clinical diagnoses as the reference standard. However, there were few negative cases with non-infectious diseases, resulting in slight bias in calculating specificity. Sun et al. assessed the performance of targeted metagenomics in diagnosing pulmonary infections in HIV-infected patients, finding an 86.7% concordance rate for the detection of main pathogens, while it was a small-sample, single-center research study, which might limit the accuracy of the study. Yang et al. investigated diagnostic value of targeted metagenomics in cancer patients with pneumonia and found its sensitivity can reach up to 84.6%. They also pointed out that the main limitation was sample size was small. Using targeted metagenomics, Zhang et al. provided a case report on *Mycobacterium bovis* infection in an infant, and patients with lung abscesses caused by *Parvimonas micra* were successfully diagnosed by Zhang et al.

Beyond pathogen detection, Zou et al. performed hybridization capture-based targeted metagenomics on patients undergoing allogeneic hematopoietic stem cell transplantation at different intervals to monitor medication efficacy, providing significant reference for treatment strategies. They also proposed that targeted metagenomics can be used to rule out infections. However, RNA viruses were considered. Shi et al. used 16S rRNA amplicon sequencing to investigate the succession of microbial communities in intensive care units treated with bacteriophage, finding that the relative abundance of target pathogens decreased while overall species diversity remained stable. Future research should focus on long-term observation of pathogen dynamics and mutations in bacterial phage receptor sites following phage treatment.

## Technical improvement of targeted metagenomics

Targeted metagenomics with high sensitivity has reduced the economic burden on patients, and its extensive application can be expected (Sun et al., 2025). Given the high sensitivity of PCR and the high throughput of mNGS, targeted metagenomics can detect pathogens with predesigned primers in the panel (Huang et al., 2023; Li et al., 2021). However, adding more primers targeting a broader range of pathogens to the panels can produce more primer dimer species, reducing the mapping rate (Xie et al., 2022) and increasing the likelihood of missing certain pathogens. Considering the epidemiology of pathogens characterized by geographical specificity (Ramirez et al., 2020), rarity (Zhan et al., 2021), and novelty (El Zein et al., 2020; Fishman, 2023), Liu et al. proposed the designing and developing regional targeted metagenomics (Xia et al., 2023; Xie et al., 2022) should be performed, and an era of widespread application of regional targeted metagenomics in diagnosing and monitoring infections with high sensitivity and low economic burden on patients can be expected.

## Challenges and limitations of targeted metagenomics

A published study explored the feasibility of capture hybridization-based targeted metagenomics and multiplex PCR-based targeted metagenomics in distinguishing lower respiratory tract infections in clinical practice. Although these methods can decrease costs with high detection ability, they have disadvantages, including long research and development cycles, limited targets, and the need to accumulate enough samples for sequencing (Yin et al., 2024). Zhao et al. provided a comprehensive review of the application of metagenomics in diagnosing infectious diseases, summarizing the advantages and disadvantages of targeted metagenomics, while health economic evaluations of metagenomics should be conducted.

## Future directions of targeted metagenomics

Given the current application scenarios, future studies can be carried out as follows:

1. Development and improvement of compatible primer pools to enhance amplification efficiency of blood samples, addressing severe nucleic acid fragmentation due to broad-spectrum antibiotics.
2. Large-scale cohort studies to evaluate host responses to different infections, determining host biomarkers for assisted diagnosis in targeted metagenomics. Combining immune repertoire analysis to characterize immunological exhaustion signatures and establishing an infection-immunity interaction model.
3. Application of syndromic panels for targeted metagenomics in diagnosing infections in blood culture, respiratory specimens, stool, and cerebrospinal fluid.
4. Technical innovation in primer design, primer dimer cleanup, turnaround time optimization, fast sample preparation, and sequencing protocols to promote quick application of targeted metagenomics in clinical settings.

## Concluding remarks

We hope that published research will inspire continued exploration and innovation, ultimately advancing our ability to detect, track, and control infectious diseases globally. We extend our gratitude to all authors who contributed their innovative work to this Research Topic. We also thank the reviewers for their rigorous and constructive feedback, which significantly enhanced the quality of the published research. Additionally, we thank the editorial team at Frontiers for their unwavering support and guidance throughout the process.

## Author disclaimer

The views expressed in this study are those of the authors and do not necessarily reflect the official policy or position of the Defense Health Agency, Department of Defense, nor the U.S. Government. This work was prepared as part of official duties. Title 17, U.S.C., Section 105 provides that copyright protection under this title is not available for any work of the U.S. Government. Title 17, U.S.C., Section 101 defines a U.S. Government work as a work prepared by a military service member or employee of the U.S. Government as part of that person’s official duties.
